# LPP slopes index attention dynamics: Evidence from mindfulness inductions

**DOI:** 10.3758/s13415-026-01457-7

**Published:** 2026-06-09

**Authors:** Ben Swanson, Yanli Lin, Matt R. Judah

**Affiliations:** Department of Psychological Science, University of Arkansas, Fayetteville, AR USA

**Keywords:** LPP slope, Late positive potential, Mindfulness

## Abstract

Substantial evidence suggests that the late positive potential (LPP) indexes motivated attention to salient stimuli. However, traditional measurement of the LPP via mean amplitude may not fully capture rapid attentional dynamics. A nascent line of work has sought to remediate this limitation by measuring the LPP slope (i.e., rate of amplitude change); yet its functional significance and sensitivity to direct experimental manipulation lacks validation. To address this gap, we re-analyzed data from a fully within-subject crossover experiment (N = 29), where attention was manipulated using focused attention (FA) and open monitoring (OM) mindfulness inductions, contrasted against an active control condition, during an affective picture viewing task involving negative and neutral images. As hypothesized, the FA induction produced flatter, more positive slopes across both negative and neutral images, suggesting enhanced attentional stability. Intriguingly, the OM induction selectively produced flatter slopes on negative but not neutral image trials, aligning with prior postulations that OM may reduce reactive disengagement from aversive stimuli. Collectively, these findings demonstrate that LPP slopes are sensitive to direct manipulations of attentional control, providing much needed experimental evidence for their utility as a neural marker of attention dynamics.

## Introduction

The late positive potential (LPP) is a widely used electrophysiological marker in affective neuroscience (Kappenman & Luck, 2011), conceptualized as a neural index of motivated attention that reflects enhanced processing of emotionally and semantically significant stimuli (Cuthbert et al., 2000; Hajcak & Foti, 2020). Characterized as a positive, sustained event-related potential (ERP) over central-parietal scalp regions, the LPP exhibits reliable amplitude enhancement in response to high, relative to low, arousing visual stimuli (Cuthbert et al., 2000; Olofsson et al., 2008). This selective sensitivity has rendered the LPP a valuable tool in diverse research areas, ranging from basic to clinical science (Hajcak et al., 2010; MacNamara, 2025; MacNamara et al., 2016). While the motivated attention framework is well-established (Hajcak & Foti, 2020), the precise functional significance of the LPP remains subject to ongoing discussion, with alternative interpretations linking its activity to broader cognitive processes such as context updating and memory encoding (Brown et al., 2012; Fields, 2023).

Irrespective of these interpretive differences, a seemingly contradictory limitation exists within the methodological conventions of quantifying the LPP. Despite its operationalization as a temporally extended response, the LPP is almost universally quantified by its mean amplitude across one or more predefined time windows (Luck, 2014). This approach, while robust, collapses an EEG signal valued for its temporal richness into voltage measured over hundreds of milliseconds relative to a baseline ending 400 + ms earlier. While this approach represents some change in the signal, it likely neglects more rapid fluctuations in the LPP occurring during a given window that may better capture the dynamic nature of attention over time.

To address this constraint, Swanson & colleagues (2025) recently advanced a novel analytic approach involving computation of LPP *slopes* as a direct means to assess rates of change in LPP amplitude. From a conceptual perspective, if LPP amplitude reflects the magnitude of allocated attention, then slopes should logically reflect the dynamics of that allocation (i.e., the extent to which attention fluctuates or stabilizes). For example, a flat slope near zero would indicate high attentional stability, whereas a steep negative or positive slope would indicate rapid change in attentional (dis)engagement across a given window. Foundationally, Swanson & colleagues (2025) established empirical support for LPP slopes as orthogonal measures to amplitude, demonstrating their psychometric reliability and capacity to explain unique variance in generalized anxiety disorder (GAD) symptoms above and beyond traditional mean amplitude and latency. Converging with this work, Monopoli et al. (2022) found that an LPP slope from 500–2,000 ms was selectively associated with unique facets of emotion dysregulation (i.e., difficulties regulating emotional escalation and de-escalation). Together, these initial studies demonstrate the promise of the LPP slope as a distinct complementary measure of affective processing.

The exact functional significance of LPP slopes remains largely theoretical. While compelling, the findings to date are fundamentally correlational, linking slope variation to individual differences (Monopoli et al., 2022; Swanson et al., 2025), without establishing sensitivity to direct experimental manipulation. If LPP slopes indeed reflect the dynamics of motivated attention, it stands to reason that they should be systematically modulated by experimental conditions that explicitly alter the deployment of attention. Consequently, the current study was designed to conduct this important test by re-analyzing data from a fully within-subject experiment that leveraged distinct mindfulness inductions to directly manipulate attentional states before and during task performance (Lin et al., 2024). 

Brief guided mindfulness inductions provide an ideal experimental paradigm for this validation. While phenomenologically complex, mindfulness is broadly defined as the nonjudgmental awareness of present-moment experience (Kabat-Zinn, 1990) and is fundamentally grounded in the cultivation of attention. We narrow investigation to focused attention (FA) and open monitoring (OM), two core yet qualitatively distinguishable mindfulness practices that have been shown to be neurocognitively differentiable and subserved via distinct attentional mechanisms (Brown et al., 2022; Fox et al., 2016; Lin, et al., 2024; Lutz et al., 2008, 2015). Briefly, FA entails sustaining attention on a target object (e.g., the breath) and redirecting attention back to the target following recognition of mind wandering. Conversely, OM is characterized by nonjudgmental, receptive awareness of experience as it arises (e.g., thoughts, feelings, bodily sensations). Consequently, FA cultivates a narrow, stable, and heightened state of attentional focus, whereas OM fosters a broad, flexible, and nondiscriminating awareness of what is experientially salient in the moment. When rigorously contrasted against an active control condition, these distinct states provide a powerful test of the functional significance of the LPP slope. The FA induction offers a direct manipulation of sustained attentional engagement, whereas the implications of the OM induction are more nuanced. Although OM does not explicitly promote sustained attention on a specific target, its cultivation of nonjudgmental awareness is nevertheless expected to alter the attentional response, particularly for aversive stimuli that often evokes reactive disengagement.

Prior research examining mindfulness effects on the LPP has generally reported a reduction in mean amplitude for negative stimuli, suggesting mindfulness promotes downregulation of emotional reactivity (Brown et al., 2013; Lin et al., 2016, 2020; Sobolewski et al., 2011; Uusberg et al., 2016; Zhang et al., 2019). However, the literature is not fully consistent; some studies report null findings or even LPP enhancement (Eddy et al., 2015; Egan et al., 2018). Moreover, standard mean amplitude measures collapse temporal data, potentially obscuring dynamic shifts in attention over time. Importantly, another central limitation of this body of work is that very few studies to date have directly contrasted FA and OM (i.e., discretizing the broader construct of mindfulness into experientially distinct states and practices), which may help to account for the mixed findings.

Accordingly, the parent study for the current investigation (Lin et al., 2024) was designed to address this gap, using a fully within-subject design to directly compare FA and OM. Intriguingly, despite the rigorous design and in contrast to a priori expectations, there were no significant main effects of the FA or OM inductions on LPP mean amplitude. Thus, this null finding using traditional LPP quantification methods directly motivated the critical question of whether LPP slopes represent a more robust and sensitive measure to mindfulness manipulations that mean amplitude.

Taken together, the present study aimed to interrogate the functional significance of LPP slopes by re-analyzing data from Lin et al. (2024). This dataset provides a critical test case, offering a natural opportunity to test whether LPP slopes are uniquely sensitive to subtle but potentially meaningful differences in attentional deployment. Specifically, we hypothesized that the FA induction, by fostering sustained attention, would produce flatter slopes (i.e., values closer to zero) across both negative and neutral image trials relative to the active control condition, reflecting enhanced attentional stability from 1000–5000 ms post picture onset. For the OM induction, we tested two competing possibilities. First, by instructing participants to monitor their internal experience (including feelings and bodily sensations) as it unfolds in response to the images, OM broadens the scope of attention during picture viewing. Rather than volitionally diverting attention away from the image as a form of active distraction, OM distributes attention processing across both the external visual stimulus and internal reactions. Thus, this widened scope of attention could theoretically result in a steeper, more negative LPP slope for salient negative images, reflecting a reduction in externalized focal attention to visual stimuli. Conversely, by promoting nonjudgmental awareness, OM could reduce the reactive tendency to disengage from aversive stimuli, which would selectively produce a flatter, more positive LPP slope on negative image trials. Confirmation of either outcome would provide critical evidence in support of the LPP slope’s functional significance, establishing it as a metric sensitive to attention dynamics.

## Method

### Design and procedures

The design and study procedures for the current study have been described elsewhere (Lin et al., 2024); we outline the protocol relevant to the current analyses here. The experiment consisted of three separate 2-h testing sessions. Each session began with participants completing a 10-min audio induction that guided them through either focused attention (FA), open monitoring (OM), or active control (C) manipulation. Participants then completed either a picture viewing or flanker task that was counterbalanced across participants. The induction manipulation (FA, OM, or control) was repeated before participants completed the remaining task.

### Participants

The final sample included 29 participants (18–35 years old, M = 20.72, SD = 4.04, 58% female) who were recruited via a variety of community sampling methods (e.g., flyers, email, word of mouth). Demographic information regarding participant race and ethnicity is available in Lin et al. (2024). The study was approved by the institutional review board (202012148) and participants were compensated $80 for completing all portions of the study.

### Tasks

#### Audio inductions

Each induction was exactly 10 min in duration. The FA induction guided participants to sustain attention on their breath and to redirect focus back to the breath upon noticing mind wandering. The OM induction instructed participants to direct awareness to present moment thoughts, feelings, and bodily sensations non-judgmentally. The active control condition consisted of listening to a TED talk that discussed how to quickly acquire second language fluency (Lonsdale, 2013). During all inductions, participants were instructed to keep their eyes open to mitigate drowsiness (Lin et al., 2020).

#### Picture viewing task

Prior to the task, participants were provided specific viewing instructions that corresponded to each induction condition. In the FA condition, participants were asked to sustain attention on presented images. In the OM condition, they were asked to nonjudgmentally attend to their internal experience as it unfolded while viewing each image. In the active control condition, participants were instructed to view each image naturally. The task consisted of 60 pictures selected from the International Affective Picture System (IAPS; Lang et al., 2008), comprising 30 high-arousing negative images and 30 low-arousing neutral images. Images were displayed via E-Prime software in three blocks. Specifically, ten negative and ten neutral images were randomly drawn from the larger stimulus set and presented randomly within each block, ensuring that image intensity and valence were balanced across blocks and testing sessions. Each trial began with a 500-ms fixation cross, followed by the image, which was displayed for 5,000 ms. Upon image offset, a blank intertrial interval was randomly jittered between 2,000 and 4,000 ms.

### EEG recording and pre-processing

Continuous EEG was recorded by using a 64-channel Lycra cap with a Brain Vision actiCHamp Plus system (Brain Vision LLC), sampling at 500 Hz from 32 electrodes placed according to the standard 10/20 system. Both vertical and horizontal electrooculogram (EOG) activity were also recorded. Offline pre-processing was performed in accordance to the procedures described in Lin et al. (2024). Data were re-referenced to the common average and filtered using a zero-phase Butterworth IIR bandpass filter from 0.01 Hz (second order, time constant ~ 15.9 s) to 20 Hz (second order). An automated algorithm identified artifacts using the following criteria: (a) a voltage step of > 50 µV between samples; (b) a difference of 400 µV in a 200-ms interval; (c) an absolute voltage difference exceeding 200 µV voltage; (d) or a difference less than 0.5 µV within a 1,000-ms interval. Trials were removed if four or more electrodes were flagged with artifacts or if any artifact was detected at a midline site (Fpz, Fz, Cz, Pz, Oz).

Consistent with the parent study, we analyzed the LPP at electrode site Pz. However, in a departure from the mean-amplitude approach, the current study analyzed the LPP continuously from 1,000–5,000 ms poststimulus onset to assess its rate of change (i.e., slope). Critically, the electrode site and time window were selected to retain exact alignment with the procedures used in the parent study (i.e., late sustained portion of the LPP at Pz; Lin et al., 2024), ensuring direct comparability. Furthermore, visual inspection of the grand-averaged waveforms confirmed that the LPP was largely linear during this time window, rendering it well-suited for slope estimation.

The cleaned waveforms were baseline corrected to 500 ms prestimulus window. Subsequently, for each participant, six averaged waveforms were computed by averaging trials within each combination of induction condition (C, FA, OM) and image valence (negative, neutral). As such, the procedure yielded 2,000 (1 observation per 2 ms aligning with the 500 Hz sampling rate) observations for each of the six waveforms (negative and neutral images for C, FA, and OM), totaling 12,000 observations per participant.

### Data analysis

To test our hypotheses, the EEG time-series data were analyzed using the nlme package in R with a restricted maximum likelihood estimator (Pinheiro et al., 2023; R core Team, 2023). Data were structured in long format, such that for every sampling point of time (i.e., 1,000 ms, 1,002 ms, etc.), there was a corresponding value for amplitude across both Condition (Control, FA, OM) and Image Valence (negative, neutral), nested within each participant. Importantly, this approach leverages the full temporal resolution of the EEG data, spanning a very large number of observations, and thus large degrees of freedom.

The primary analysis modeled the three-way interaction between Time, Condition (Control, Focused Attention, Open Monitoring) and Image Type (Negative, Neutral), with a random intercept fitted for each participant. Consequently, the coefficient for Time and its respective interactions represent the LPP slope or the rate of amplitude change per unit of time. As such, the estimated coefficient for time is referred to as the LPP slope below. The full model can be expressed in Wilkinson Notation as:

$$\begin{array}{l}Amplitude\sim Time+Condition+Image\;Valence+Time\ast Condition+Time\ast Image\;Valence\\+\;Condition\ast Image\;Valence+Time\ast Condition\ast Image\;Valence+\left(1\left|Participant\right.\right)\end{array}$$

Both Condition (C, FA, OM) and Image Valence (negative, neutral) were dummy coded such that control and neutral conditions served as the reference groups, enabling direct statistical comparison between each mindfulness induction and the active control, and negative to neutral trials, respectively.

#### Psychometric validation

Given that LPP slope measurement is relatively underexplored compared to more traditional LPP measures, we conducted several additional analyses to determine the extent to which the slope may have been associated with other LPP features. For instance, it is reasonable to question whether LPP slopes reflect an overlapping attentional process already captured by mean amplitude and latency. To assess this directly, participant level slopes were estimated within each combination of Image Valence (Negative, Neutral) for each Condition (C, FA, OM) using a linear model. Specifically, Time was regressed on Amplitude for each participant level waveform across the six possible combinations of Image Valence and Condition. The coefficient for Time (i.e., the slope) was then extracted. Mean amplitude was calculated as the mean amplitude value from 1,000 to 5,000 ms. Given the LPP peaked at approximately 500 ms across conditions, peak latency was calculated as the time where amplitude was maximal anywhere from 350 to 650 ms.

In addition to traditional measurement techniques, we also examined several additional LPP measures we had reason to believe might influence slope measurement. First, we examined whether activity preceding the measurement window, hence referred to as pre-slope voltage, was related to LPP slopes (Swanson et al., 2025). Indeed, a relatively large pre-slope voltage might be expected to correlate with a more subsequently negative slope. A large correlation between LPP slope and pre-slope voltage might suggest the LPP slope may not reflect attentional dynamics but rather captures amplitude regressing to the mean. As such, we extracted amplitude value at 998 ms for each participant within each image valence (negative and neutral) and condition (C, FA, OM). Second, we conducted additional analyses to assess whether variability in latency across trials could have influenced slope measurement. Specifically, it was possible that greater trial level variability in peak amplitude may have influenced the later measured slope. To assess this consideration, peak latency values were extracted from single trial data. Specifically, we extracted the time at which amplitude was maximal from 350–650 ms for each trial. Lastly, we calculated the standard deviation of single trial latencies within each combination of Condition (C, FA, OM) and Image Valence (Negative and Neutral) for each participant. This standard deviation of the extracted latencies served as our metric for latency variability.

The calculated LPP measures allowed us to examine whether LPP slopes indeed reflected a dissociable measure of attention. LPP slopes, mean amplitude, latency, pre-slope voltage, and latency variability were z-scored within each combination of Condition (C, FA, OM) and Image Valence (Negative, Neutral) for a total of six observations for each participant. We then examined bivariate correlations between LPP slope and mean amplitude, latency, pre-slope voltage, and latency variability.

## Results

**Fig. 1:**
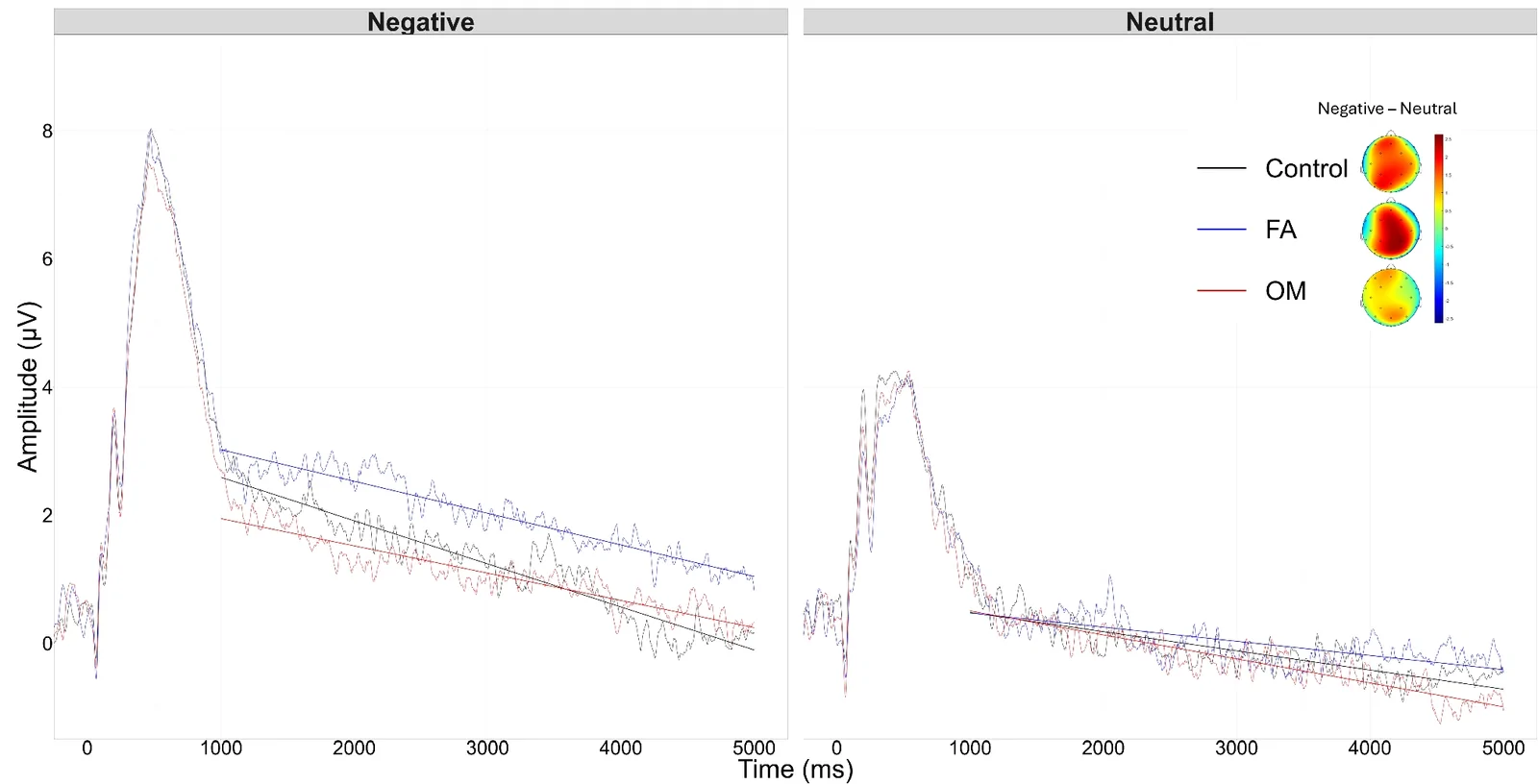
Grand average waveform. *Note.* The grand average waveform is shown. The regression lines displayed were calculated directly from the model reported in Table 1

Our primary model tested the effects of the mindfulness inductions on LPP slopes across the 1000–5000 ms time window. Full model results are presented in Table 1 and a visual depiction of the model is in Fig. 1. The model revealed a significant three-way interaction involving Time (i.e., LPP slope), Condition, and Image Valence for both the OM, *t*(347,960) = 16.70, *p* <.001, and FA, *t*(347,960) = 5.22, *p* <.001, induction relative to the active control. These results indicate that the effect of image valence (negative vs. neutral) on the LPP slope was significantly different during both the OM and FA induction relative to the control condition. To further parse this interaction, we conducted separate follow-up analyses for negative and neutral image trials (Table 2).

**Table 1:** Fixed effects of multilevel model

	*b*	*SE*	*df*	*t*	*p*
(Intercept)	0.48	0.32	347,960	1.50	.134
Time	− 0.0006	0.00002	347,960	− 31.10	<.001
FA	− 0.01	0.03	347,960	− 0.24	.809
OM	0.02	0.03	347,960	0.76	.445
Negative Image	2.11	0.03	347,960	66.91	<.001
Time:FA	0.0002	0.00003	347,960	5.74	<.001
Time:OM	− 0.0002	0.00003	347,960	− 5.50	<.001
Time:Negative Image	− 0.0007	0.00003	347,960	− 27.38	<.001
FA:Negative Image	0.44	0.04	347,960	9.89	<.001
OM:Negative Image	− 0.66	0.04	347,960	− 14.90	<.001
Time:FA:Negative:Image	0.0002	0.00004	347,960	5.22	<.001
Time:OM:Negative:Image	0.0006	0.00004	347,960	16.70	<.001

*b* represents the unstandardized regression coefficient. The estimated coefficient for time represents the LPP slope. Because the data was recorded at the 500 Hz sampling rate, coefficients involving Time (i.e., LPP slope) reflect the estimated change in LPP amplitude (in µV) for every 2-ms increment. Image valence was dummy coded such that 0 = neutral, and 1 = threat. Furthermore, Condition was dummy coded such that the control condition served as the reference group

**Table 2:** Fixed effects of multilevel model

	*b*	*SE*	*df*	*t*	*p*
*Negative Images*					
(Intercept)	2.58	0.43	173,966	6.02	<.001
Time	− 0.001	0.00001	173,966	− 70.15	<.001
FA	0.43	0.03	173,966	13.81	<.001
OM	− 0.64	0.03	173,966	− 20.40	<.001
Time:FA	0.0004	0.00003	173,966	13.18	<.001
Time:OM	0.0005	0.00003	173,966	18.20	<.001
*Neutral Images*					
(Intercept)	0.48	0.32	179,966	1.48	.14
Time	− 0.0006	0.00002	179,966	− 37.70	<.001
FA	− 0.008	0.03	179,966	− 0.29	.769
OM	0.02	0.03	179,966	0.93	.355
Time:FA	0.0002	0.00002	179,966	6.96	<.001
Time:OM	− 0.0002	0.00002	179,966	− 6.67	<.001

Image valence was coded such that 0 = neutral, and 1 = threat. Furthermore, Condition was dummy coded such that the control condition served as the reference group. The estimated coefficient for time represents the LPP slope

Considering only negative trials, both mindfulness inductions modulated the LPP slope compared to the control condition, as indicated by significant Time × Condition interactions. Specifically, the LPP slope was flatter (i.e., more positive) during both FA, *t*(173,966) = 13.18, *p* <.001, and OM, *t*(173,966) = 18.20, *p* <.001, compared with the control condition. The model also revealed that LPP amplitude at the intercept of Time was greater for FA, *t*(173,966) = 13.81, *p* <.001, and smaller for OM, *t*(173,966) = − 20.40, *p* <.001, relative to the control condition. These results suggest that, during negative images, both FA and OM were associated with flatter, more positive LPP slopes over time.

**Fig. 2:**
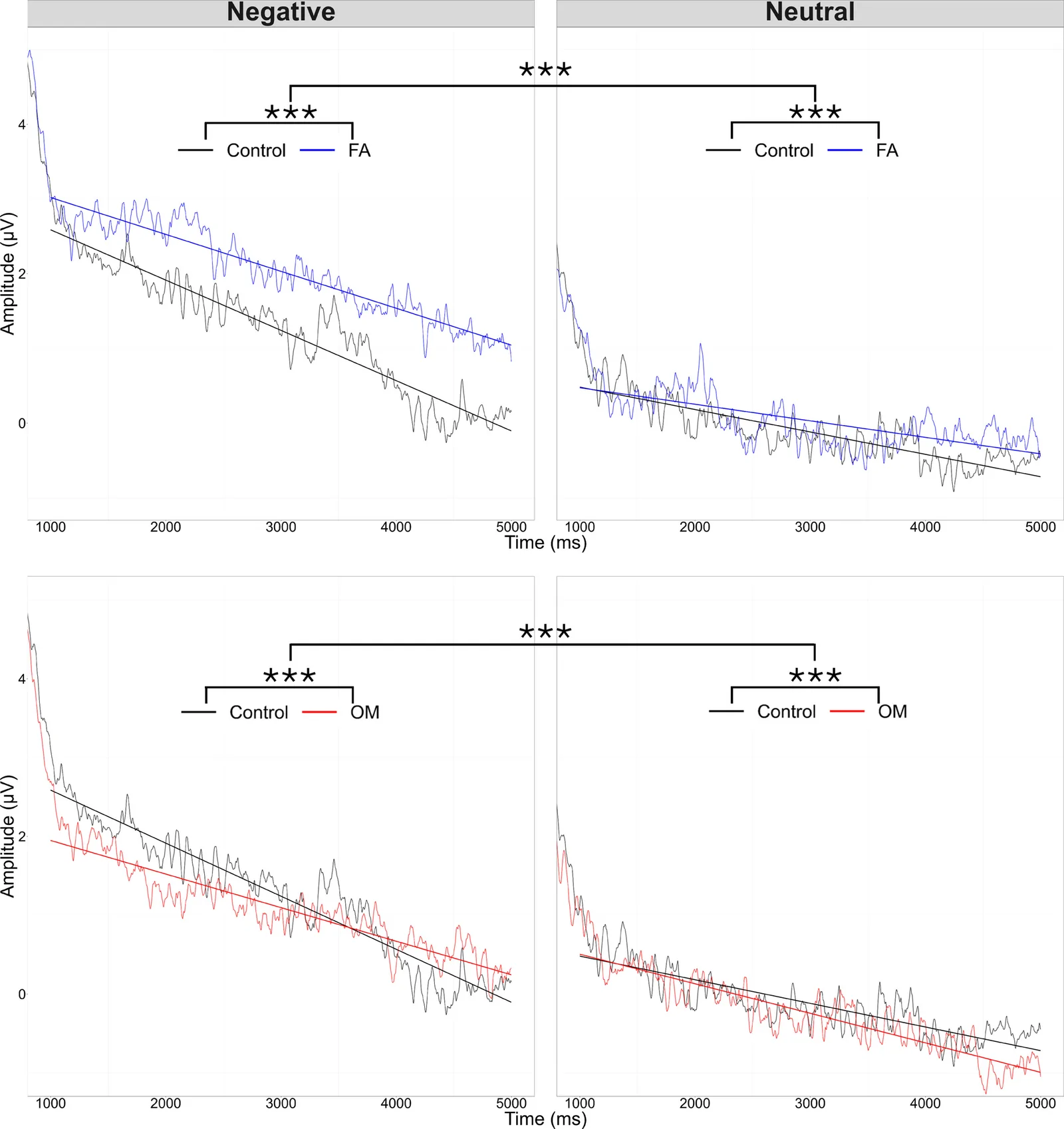
Slope comparisons. *Note.* The figure reflects the comparisons from the main analysis. Overall, FA and OM demonstrated flatter, more positive slopes compared to controls in all conditions except neutral open monitoring trials. The regression lines shown were calculated directly from the negative and neutral models presented in Table 2

Within neutral trials only, the FA induction continued to produce flatter, more positive slopes compared to the control condition, *t*(173,966) = 6.96, *p* <.001. Conversely, the slope was steeper and more negative during the OM induction compared to the control condition, *t*(173,966) = − 6.67, *p* <.001. The LPP amplitude at the intercept of Time for FA, *t*(173,966) = − 0.29, *p* =.769, and OM, *t*(173,966) = 0.93, *p* =.355, did not differ relative to the control condition. Taken together, the FA induction produced flatter slopes across both negative and neutral image trials, whereas the OM induction was selectively associated with flatter slopes only on negative but not neutral image trials. Thus, flatter slopes during negative images and steeper slopes during neutral images were responsible for driving the three-way interaction observed in the full factorial model in the OM condition (Fig. 2). For the FA condition, slopes were flatter relative to the control condition in both negative and neutral images.

### Sensitivity analyses

To validate the LPP slope as a distinct metric, we examined its relationship with traditional measures and potential confounds. Bivariate correlations revealed that LPP slopes were weakly correlated with both mean amplitude,* r* =.17, *p* =.022 and peak latency,* r* = −.17, *p* =.024, and pre-slope voltage, *r* = −.27, *p* <.001. Moreover, LPP slopes were not correlated with latency variability,* r* = −.06, *p* =.444. Together, these findings suggest LPP slopes may capture a feature of attentional dynamics that is distinct from standard mean amplitude or timing variability.

## Discussion

Our findings provide initial experimental evidence that LPP slopes are a sensitive index of attentional dynamics. As hypothesized, applying focused attention to both negative and neutral images resulted in a flatter slope compared to a control condition, suggesting that LPP slopes index the stability of attentional engagement. Specifically, by demonstrating that volitional focus on external visual stimuli sustains the LPP amplitude and thereby flattens its slope, our study offers LPP slopes as a candidate marker from which to interrogate and refine the functional significance of this emerging neurophysiological measure.

Critically, our results build on a growing literature investigating LPP slopes. Previous studies have linked the slope to individual differences, such as GAD symptomology and self-reported emotion dysregulation (Monopoli et al., 2022; Swanson et al., 2025). While foundational, these studies did not directly manipulate attention. If LPP amplitude reflects attention, then it stands to reason that the rate of change in amplitude (i.e., the slope) should ostensibly reflect the dynamics of attention allocation. Our findings provide support for this reasoning, showing that the rate of LPP reduction (i.e., the steepness of the downward slope) is flattened by focused attention, potentially indicating greater overall attentional stability. Indeed, this interpretation aligns with extant neurobiological accounts of the LPP as an attention orienting response driven by the locus coeruleus norepinephrine system (Hajcak & Foti, 2020).

Adjudicating the competing possibilities for open monitoring, the OM induction produced a flatter LPP slope on negative but showed the opposite pattern on neutral trials. This finding is not only consistent with the longstanding proposition that nonjudgmental awareness attenuates emotional reactivity to aversive stimuli, but it also clearly demonstrates that this may be achieved via sustained attention to the triggering stimulus, rather than broader distribution of attention processing across internal and external states. Building upon prior work linking mindfulness, including the OM state in particular, to reduced LPP *mean amplitudes* to negative images (Brown et al., 2012; Lin et al., 2016, 2020; Sobolewski et al., 2011; Uusberg et al., 2016), our slope analysis sheds light on the underlying attentional *dynamic*, providing new evidence that OM fosters a state of receptive, stimulus-driven attention, resulting in greater attentional stability and engagement to emotionally evocative images relative to low-salience neutral stimuli. The flatter LPP slope on negative but steeper LPP slope on neutral images in the OM condition may also suggest OM increases attentional breadth more broadly. As such, our interpretation that OM cultivates receptive, stimulus-driven attention is a plausible, but not definitive, explanation of the current findings.

Our findings of both convergent (flatter slopes for negative images) and divergent (different slope directions on neutral images) contribute to the growing literature highlighting the neurophysiological and functional distinctions between FA and OM. For example, both forms of meditation have been shown to reduce functional connectivity between the striatum and posterior cingulate cortex, a default mode network hub (Fujino et al., 2018). However, they can exert opposing effects on the functional connectivity between the ventral striatum and the visual retrosplenial cortices, with FA increasing functional connectivity and OM reducing it. In line with its established link to executive function, FA has been found to increase sustained attention (Brefczynski-Lewis et al., 2007; Carter et al., 2005; Lippelt et al., 2014) and modulate the default-mode, salience, and executive control networks, key brain hubs involved in attention processing (for a review see Ganesan et al., 2022).

Recent direct comparisons further underscore these distinctions. Brown & colleagues (2022) found that FA, but not OM, uniquely increased left hemispheric alpha, a neural correlate of approach motivation, which has intriguingly been shown to narrow attention (Gable & Harmon-Jones, 2008). In contrast, another recent comparative study showed that relative to FA, OM produced elevated autonomic arousal but reduced salivary cortisol (Ooishi et al., 2021), in line with our postulation that the regulatory properties of OM may reflect a selective willingness to engage with evocative stimuli or experiences. Taken together, our results complement this body of work by demonstrating that FA and OM involve distinct attentional dynamics during visual affective processing, uniquely identifiable via LPP slopes.

While our main findings suggested that LPP slopes captured distinct attentional dynamics, a question that remained was whether LPP slopes captured attentional dynamics dissociable from other LPP measures. Specifically, whether slopes correspond with other LPP features (e.g., amplitude, latency, pre-slope voltage, latency variability) is an important question addressed by our paper. If LPP slopes were to strongly correlate with these LPP measures, it might suggest that slopes may not reflect stability in attention over time. Importantly, LPP slopes were only weakly correlated with both mean amplitude and latency. LPP slopes were also weakly correlated with pre-slope voltage, while latency variability was not correlated with LPP slopes. These findings suggest that the LPP slopes we measured represent both a conceptual and psychometrically dissociable measure of the attentional response. Taken together with the experimental effects on the slopes, our findings suggest that LPP slopes may capture subtle changes in attentional (dis)engagement.

One of the motivating factors for the current study was to compare results of the LPP slopes analysis with prior mean-amplitude results from the parent study (Lin et al., 2024). The parent study found no condition differences across OM and FA when examining LPP mean amplitudes. However, our analysis of LPP slopes revealed dissociable effects across both FA and OM. These results provide initial evidence that LPP slopes carry utility as a temporally sensitive index of attentional processing. Furthermore, because the parent study found that mean amplitude differences emerged only after accounting for self-reported arousal, it is possible that LPP slopes are similarly influenced by participants' subjective reactions to images. We chose not to examine this question directly given the practical and conceptual challenges of fitting and interpreting four-way interactions. Indeed, the nascent literature on LPP slopes would benefit from additional studies to develop methodological best practices to probe such questions.

Despite strengths of the current study, several limitations should be considered in interpreting our findings. Although we used a fully within-subjects design, the sample size was modest and thus future studies, including replication efforts, involving larger samples are encouraged. Our study also relied on guided inductions and instructional manipulations; thus, the degree to which participants successfully adopted and maintained the intended states remain fundamentally unclear, although this is typical of many LPP designs (Hajcak & Foti, 2020; Lin, et al., 2024). Relatedly, given that the inductions were administered prior to task performance, it is possible that the strength of these states decayed over time. Thus, future research utilizing longer tasks may benefit from modeling block-level effects to explicitly investigate the temporal stability or potential washout of induced mindfulness states. Furthermore, the selection of a measurement window from 1,000 to 5,000 ms was justified by our goal of understanding attention dynamics over a sustained period that matched the parent study to enable direct comparison (Lin et al., 2024), rather than the initial orienting response. However, this approach ignores earlier and faster LPP dynamics observed in prior studies (Monopoli et al., 2022; Swanson et al., 2025). Our use of a noncausal low-pass filter was well-suited to preserving peak latency (Zhang et al., 2024) by removing phase-induced time delays; however, it did so at the cost of smearing ERP onset earlier (Rousselet, 2012). Future studies are needed to understand how filter selection affects ERP slopes. Ultimately, our findings argue in favor of continuing research to better understand the functional significance of LPP slopes and how they are affected by cognitive and affective manipulations.

In summary, our findings provide critical experimental support for LPP slopes as a unique indicator of attentional dynamics. Specifically, our results suggests that a flatter slope in the 1,000–5,000 ms window may reflect sustained attentional stability to a visual stimulus, whether achieved through the top-down “broadband” deployment of focused attention or through the bottom-up salience driven engagement of open monitoring. In addition to supporting the interpretation of prior LPP studies and validating the use of the LPP slopes as a meaningful neurophysiological measure, our findings contribute to a more nuanced understanding of distinct mechanisms underlying different mindfulness states and practices.

## Data Availability

Data or materials are available upon request.

## Code availability

Code used to conduct the analysis is available upon request.
